# Effects of onabotulinumtoxinA treatment in chronic migraine patients with and without daily headache at baseline: results from the COMPEL Study

**DOI:** 10.1186/s10194-018-0953-0

**Published:** 2019-02-01

**Authors:** William B. Young, J. Ivan Lopez, John F. Rothrock, Amelia Orejudos, Aubrey Manack Adams, Richard B. Lipton, Andrew M. Blumenfeld

**Affiliations:** 1 Jefferson Hospital for Neuroscience, 900 Walnut Street, Second Floor, Suite #200, Philadelphia, PA 19107 USA; 2 University of South Alabama College of Medicine, Mobile, AL USA; 3 George Washington School of Medicine, Washington, DC USA; 4 Allergan plc, Irvine, CA USA; 5 Montefiore Headache Center, Department of Neurology, Department of Epidemiology and Population Health, Albert Einstein College of Medicine, Bronx, NY USA; 6 Headache Center of Southern California, The Neurology Center, Carlsbad, CA USA

**Keywords:** COMPEL, Daily headache, Disability, Migraine, OnabotulinumtoxinA, Quality of life

## Abstract

**Background:**

OnabotulinumtoxinA is effective in preventing chronic migraine (CM); however, the benefit of onabotulinumtoxinA in patients with CM with daily headache is unknown because these patients are typically excluded from clinical trials. This subanalysis of the COMPEL Study assessed the efficacy and safety of onabotulinumtoxinA in people with CM with and without daily headache.

**Methods:**

In total, 715 patients received onabotulinumtoxinA 155 U with or without concomitant oral preventive treatment. Patients who had complete daily diary records for the 28 days of the baseline period were stratified based on daily headache status. The primary outcome variable was reduction in headache-day frequency per 28-day period at 108 weeks (after 9 treatment cycles) relative to baseline. Exploratory outcomes included moderate to severe headache days, migraine disability (using the Migraine Disability Assessment [MIDAS] questionnaire), and health-related quality of life (Migraine-Specific Quality-of-Life Questionnaire v2 [MSQ]). Adverse events and their relatedness were recorded.

**Results:**

Overall, 641 patients had complete daily diary records at baseline. In patients with daily headache (*n* = 138) versus without (*n* = 503), treatment with onabotulinumtoxinA was associated with a significant mean (SD) reduction in 28-day headache-day frequency relative to baseline at week 108 (− 10.5 [9.2] vs − 12.2 [6.7], respectively; both *P* < 0.001) with no significant between-group difference (*P* = 0.132). The mean (SD) reduction in moderate to severe headache days at week 108 was significant in patients with and without daily headache (− 11.5 [9.4] and − 9.9 [6.4]; *P* < 0.001) with no significant between-group difference (*P* = 0.153). Mean (SD) MIDAS scores significantly improved from baseline at week 108 (− 43.3 [73.4] and − 43.6 [46.7]; both *P* < 0.001), with no significant between-group difference (*P* = 0.962). Similarly, mean (SD) MSQ subscale scores significantly improved from baseline at week 108 for patients with and without daily headache. OnabotulinumtoxinA was well tolerated in patients with and without daily headache.

**Conclusion:**

Results indicate that onabotulinumtoxinA is associated with reductions from baseline in headache-day frequency and improvements in disability and quality of life for up to 108 weeks in people with CM with daily headache; however, a longer duration of treatment was required to fully realize the treatment effect on headache. No new safety concerns were identified.

**Electronic supplementary material:**

The online version of this article (10.1186/s10194-018-0953-0) contains supplementary material, which is available to authorized users.

## Introduction

Chronic migraine (CM) is a debilitating disease occurring in 1.4% to 2.2% of adults globally [1]. CM varies among individuals in pain intensity, headache-day frequency, allodynia, and overall migraine-related disability. It is recognized that people with concomitant diseases and more headache-related characteristics may respond to treatment differently than those without these factors [2]. It is well recognized in clinical practice that the preventive treatment of daily headache (including daily headache attacks associated with CM) is challenging [3]. For example, people with CM and chronic daily headache of ≥6 months’ duration have been shown to be more likely to be nonresponders to topiramate preventive treatment than those without chronic daily headache [4]. Furthermore, daily headache can be complex; those with more severe manifestation such as unremitting headache (headache > 80% of waking time) or CM with continuous pain (no pain-free periods during screening) are often excluded from clinical trials for preventive treatment [5, 6].

The efficacy and safety of onabotulinumtoxinA for the prevention of CM was first established in the double-blind, placebo-controlled Phase III REsearch Evaluating Migraine Prophylaxis Therapy (PREEMPT) trials [7–9]. The open-label extension phase of PREEMPT further confirmed the efficacy and safety of onabotulinumtoxinA over an additional 32 weeks [10]. The Chronic migraine OnabotulinuMtoxinA Prolonged Efficacy open-Label (COMPEL) Study was subsequently undertaken to gather real-world evidence on the long-term management of CM, evaluating efficacy and safety data on onabotulinumtoxinA after 9 treatments (108 weeks) [11, 12]. The COMPEL Study also enrolled patients with more complex CM and sought to determine the benefit of onabotulinumtoxinA in these patient groups. We undertook this analysis of the COMPEL Study to compare the efficacy and safety of onabotulinumtoxinA in patients with CM with and without daily headache at baseline.

## Methods

### Primary study design

The COMPEL Study was an open-label, prospective study in adults with CM undertaken across multiple sites in the United States, Australia, and South Korea (ClinicalTrials.gov identifier NCT01516892). The methodology of the COMPEL Study has been published [11] and will be briefly reviewed here. OnabotulinumtoxinA (BOTOX®; Allergan plc, Dublin, Ireland) 155 U was administered every 12 weeks for 9 treatment cycles (108 weeks) [11] using the fixed-site, fixed-dose injection paradigm [13]. Adult patients aged ≥18 years with a diagnosis of CM and with stable comorbidities who had not previously received onabotulinumtoxinA were eligible for enrollment. Patients could be taking a stable oral preventive treatment at baseline. We excluded patients if they were pregnant or planning a pregnancy or if they had severe major depressive disorder or suicidal ideation. We obtained ethical approval from the institutional review board or independent ethics committee at each study site and written informed consent from all patients before study enrollment.

The primary outcome measure was the change from baseline in headache days per 28-day period at 108 weeks (after 9 treatment cycles) [11]. The secondary outcome measures were the mean changes from baseline in headache days at week 60 (after 5 treatment cycles) and in the 6-item Headache Impact Test (HIT-6) total score over a 4-week period at weeks 60 and 108. Exploratory outcome measures included, but were not limited to, assessment of the change from baseline in moderate to severe headache days; HIT-6 scores throughout the study; migraine-related disability as measured by Migraine Disability Assessment Questionnaire (MIDAS) scores, with higher scores indicating greater disability [14]; and health-related quality of life as measured by the Migraine-Specific Quality-of-Life Questionnaire v2 (MSQ) scores, with higher scores indicating greater quality of life [15]. Moderate to severe headache days were assessed via the patient’s daily diary, and HIT-6 and MIDAS were assessed at each clinic visit. The MSQ was assessed at baseline and at weeks 48, 96, and 108.

Safety and tolerability were assessed for all patients who received ≥1 onabotulinumtoxinA treatment. Patients were withdrawn from the study if they showed any signs of suicidal ideation or became pregnant.

### Subgroup analysis

A subpopulation with and without daily headache was assessed. The daily headache and no daily headache groups included only patients who had diary entries every day of the 28-day screening period and had headache on all 28 days (daily headache) or < 28 days (no daily headache) at baseline.

### Statistical analysis

As previously described, for primary and secondary analyses, including change from baseline in headache days and HIT-6 scores, missing data were imputed using a modified last-observation-carried-forward (mLOCF) methodology [12]. The exploratory analyses reported here in patients with and without daily headache (headache-day frequency, patient-reported outcomes, and safety) were descriptive and inferential, characterizing trends associated with onabotulinumtoxinA treatment over 108 weeks and using observed data only. The change from baseline for each subgroup (ie, those with and without daily headache) was assessed in patients with baseline and visit data for the specific time point being assessed; *P* < 0.05 was considered statistically significant. The difference between subgroups (ie, between those with and those without daily headache at baseline) was assessed using 2-sided *t* tests (alpha = 0.05).

## Results

### Patient demographics and disposition

We enrolled 716 patients (safety population); of these, 715 received ≥1 dose of onabotulinumtoxinA (analysis population). Of the 715 patients in the analysis population, 641 had complete diary data for the 28 days of the screening phase. Of these 641 patients, 138 (21.5%) met study criteria for daily headache at baseline.

Demographics at baseline were similar in patients with and without daily headache, with the exception of sex (Table 1). There were fewer women with daily headache (72.5%) than without daily headache (88.9%). Clinical characteristics were generally similar across subgroups, with the exception of family history of migraine (Table 1). Compared with patients without daily headache at baseline, patients with daily headache had a greater number of mean (SD) headache days (28.0 [0.0] vs 20.3 [4.0]) and moderate to severe headache days (24.1 [5.1] vs 16.4 [4.5]) at baseline and were less likely to have a family history of migraine (57.3% vs 64.6%).

**Table 1:** Demographics and Baseline Clinical Characteristics for Patients With and Without Daily Headache

Characteristic	With Daily Headache (*n* = 138)	Without Daily Headache (*n* = 503)
Age, mean (SD), y	42.5 (11.7)	43.3 (10.9)
Min, max	18, 71	18, 72
Female, n (%)	100 (72.5)	447 (88.9)
Race, n (%)		
Caucasian	116 (84.1)	417 (82.9)
Black or African American	7 (5.1)	32 (6.4)
Asian	14 (10.1)	51 (10.1)
Native Hawaiian or other Pacific Islander	1 (0.7)	2 (0.4)
American Indian or Alaska Native	0 (0.0)	1 (0.2)
BMI, kg/m^2^, mean (SD)	28.0 (6.6)	27.5 (6.6)
Age of migraine onset, mean (SD), y	32.7 (14.7)	32.3 (13.6)
Time since onset of migraine, mean (SD), y	9.9 (10.7)	11.1 (11.3)
Family history of migraine, n (%)	79 (57.3)	325 (64.6)
Headache days at baseline, mean (SD)	28.0 (0.0)	20.3 (4.0)
Moderate/severe headache days at baseline, mean (SD)	24.1 (5.1)	16.4 (4.5)
Medication use at baseline, n (%)^a^		
Previously taken acute medications	136 (98.6)	499 (99.2)
Previously taken preventive medications	114 (82.6)	413 (82.1)

^a^ Data based on safety population

*BMI* body mass index

Among all patients (*N* = 716), 373 (52.1%) completed the study. The most common reasons for study discontinuation were withdrawal of consent (*n* = 92 [12.8%]), lost to follow-up (*n* = 82 [11.5%]), lack of efficacy (*n* = 25 [4.9%]), and adverse events (AEs; *n* = 25 [3.5%]). Sixty of the 138 patients (43.5%) with daily headache completed the study compared with 277 of the 503 patients (55.1%) with no daily headache.

Of the patients with daily headache, a cumulative total of 24 (17.4%) patients discontinued after treatment 2, 50 (36.2%) after treatment 5, and 78 (56.5%) after the final treatment. Of the patients without daily headache, a cumulative total of 89 (17.7%) patients discontinued after treatment 2, 157 (31.2%) after treatment 5, and 227 (45.0%) after the final treatment.

### Efficacy outcomes

Overall efficacy outcomes have been published [12] and are reviewed briefly for context. In the analysis population of 715 patients, headache days were reduced from the first assessment (at week 24 after 2 treatment cycles), and reductions continued throughout the 108-week period. By week 108 (after 9 treatment cycles), onabotulinumtoxinA had significantly reduced mean (SD) headache-day frequency (− 10.7 [6.4] days from baseline; *P* < 0.0001) and mean (SD) HIT-6 scores (− 7.1 [7.2] from baseline; *P* < 0.0001).

Of the 282 patients who completed the study and had headache-day data from all 5 study visits (including baseline), onabotulinumtoxinA was associated with a slightly greater reduction in mean (SD) headache days from baseline (− 11.8 [7.3] days) compared with the overall analysis population (− 10.7 [6.4] days).

#### Subgroup of patients with and without daily headache

##### Effect on headache-day frequency

**Fig. 1:**
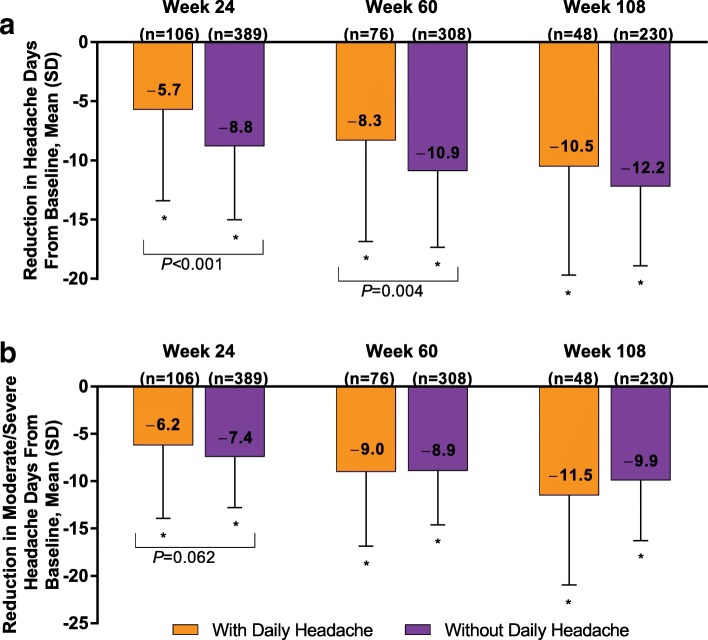
OnabotulinumtoxinA effect on (**a**) headache-day frequency and (**b**) moderate to severe headache-day frequency. *P < 0.001 for within-treatment-group comparison from baseline. *P* values shown in the figure are between-subgroup differences in change from baseline; data are observed data

At baseline, patients with daily headache had a mean (SD) of 28.0 (0.0) headache days compared with 20.3 (4.0) headache days in patients with no daily headache. OnabotulinumtoxinA significantly reduced the mean (SD) frequency of headache days per 28-day period in patients with versus without daily headache at baseline to 22.3 (7.7) and 11.8 (6.9), respectively, at week 24; to 19.7 (8.6) and 9.5 (6.4) days at week 60; and to 17.5 (9.2) and 8.1 (6.7) at week 108 (all *P* < 0.001 for within-group comparisons with baseline; Additional file 1: Figure S1A). The mean (SD) change from baseline in headache days was − 5.7 (7.7) and − 8.8 (6.2), respectively, at week 24; − 8.3 (8.6) and − 10.9 (6.4) days at week 60; and − 10.5 (9.2) and − 12.2 (6.7) days at week 108 (Fig. 1a). There was a statistically significant mean between-group difference in the change in headache-day frequency from baseline at weeks 24 (3.2; *P* < 0.001) and 60 (2.6; *P* = 0.004) but not week 108 (primary efficacy endpoint; 1.7; *P* = 0.132). A total of 18 of 106 patients (17.0%) with daily headache at baseline had a ≥ 50% reduction in headache frequency from baseline at week 24; 19 of 48 patients (39.6%) with daily headache at baseline had a ≥ 50% reduction in headache frequency at week 108.

Similarly, onabotulinumtoxinA significantly reduced mean (SD) moderate to severe headache days in patients with and without daily headache to 17.8 (8.5) and 9.1 (5.9) days, respectively, at week 24; to 15.1 (8.6) and 7.3 (5.3) days at week 60; and to 12.2 (8.4) and 6.2 (5.9) days at week 108 (a change of − 11.5 [9.4] and − 9.9 [6.4]; all *P* < 0.001 for within-group comparisons with baseline; Additional file 1: Figure S1B). The mean (SD) change from baseline in moderate to severe headache days was − 6.2 (7.7) and − 7.4 (5.4), respectively, at week 24; − 9.0 (7.9) and − 8.9 (5.7) at week 60; and − 11.5 (9.4) and − 9.9 (6.4) at week 108 (Fig. 1b). There was no significant between-group difference in the change from baseline at any time point.

##### Patient-reported outcomes

**Fig. 2:**
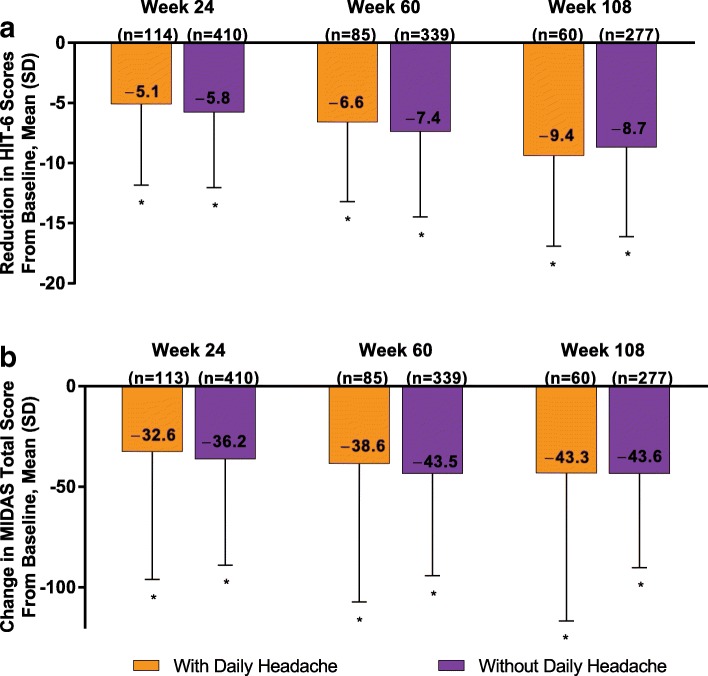
OnabotulinumtoxinA effect on (**a**) HIT-6 scores and (**b**) MIDAS scores. HIT-6 = 6-Item Headache Impact Test; MIDAS = Migraine Disability Assessment. *P < 0.001 for within-treatment-group comparison from baseline; data are observed data

OnabotulinumtoxinA significantly reduced mean (SD) HIT-6 total scores from a baseline of 65.6 (5.2) and 64.6 (4.6) in patients with and without daily headache at baseline, respectively, to 60.9 (7.1) and 58.6 (6.8) for the 28-day period before week 24; to 59.6 (6.7) and 56.8 (7.1) at week 60; and to 55.9 (7.7) and 55.3 (7.5) at week 108 (all *P* < 0.001 for within-group comparison with baseline). The mean (SD) change in HIT-6 scores from baseline for patients with and without daily headache were − 5.1 (6.8) and − 5.8 (6.2), respectively, at week 24; − 6.6 (6.6) and − 7.4 (7.1) at week 60; and − 9.4 (7.5) and − 8.7 (7.4) at week 108 (Fig. 2a). There was no significant between-group difference in the reduction in HIT-6 from baseline at any time point from week 24.

OnabotulinumtoxinA significantly reduced mean (SD) MIDAS total scores in patients with and without daily headache from a baseline score of 90.6 (66.3) and 73.3 (53.4), respectively, to 63.7 (66.3) and 35.5 (43.3) at week 24; to 56.2 (68.2) and 25.4 (30.8) at week 60; and to 41.9 (60.1) and 20.5 (28.1) at week 108 (all *P* < 0.001 for within-group comparison with baseline). The mean (SD) change from baseline for patients with and without daily headache was − 32.6 (63.4) and − 36.2 (52.7), respectively, at week 24, − 38.6 (68.6) and − 43.5 (50.8) at week 60, and − 43.3 (73.4) and − 43.6 (46.7) at week 108 (Fig. 2b). There was no significant between-group difference in change from baseline at any time point, including week 108 (mean between-group difference, 0.4; *P* = 0.962).

**Fig. 3:**
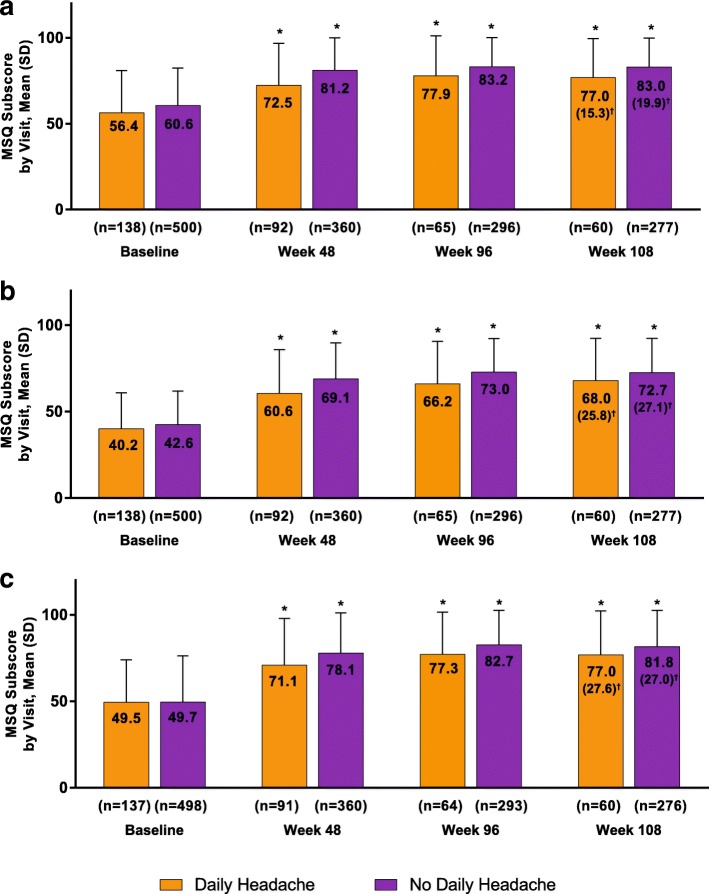
OnabotulinumtoxinA effect on MSQ Role Functions: (**a**) Preventive, (**b**) Restrictive, and (**c**) Emotional Function subscores. MSQ = Migraine-Specific Quality-of-Life Questionnaire. *P < 0.001 for within-treatment-group comparison from baseline; data are observed data. ^†^Change from baseline

Similarly, MSQ domain scores were significantly increased (improved) at all time points compared with baseline, regardless of patient daily headache status at baseline (Fig. 3). Mean (SD) MSQ Role Function Preventive scores increased from a baseline of 56.4 (24.5) and 60.6 (21.8) for patients with and without daily headache, respectively, to 72.5 (24.3) and 81.2 (18.8) at week 48 and to 77.0 (22.5) and 83.0 (16.9) at week 108 (all *P* < 0.001 for within-group comparison with baseline). The mean (SD) change from baseline for patients with and without daily headache was similar at week 48 (17.4 [23.2] and 19.1 [20.3], respectively) and at week 108 (15.3 [28.0] and 19.9 [19.9]; Figs. 3a and b). Mean (SD) MSQ Role Function Restrictive scores increased from a baseline of 40.2 (20.7) and 42.6 (19.3) for patients with and without daily headache, respectively, to 60.6 (25.4) and 69.1 (20.6) at week 48 and to 68.0 (24.5) and 72.7 (19.7) at week 108 (all *P* < 0.001 for within-group comparisons with baseline). The mean (SD) change from baseline for patients with and without daily headache increased slightly from week 48 (21.5 [25.8] and 24.7 [21.6], respectively) to week 108 (25.8 [26.1] and 27.1 [22.3]; Fig. 3a and b). Mean (SD) MSQ Emotional Function scores significantly increased from a baseline of 49.5 (24.6) and 49.7 (26.5) for patients with and without daily headache, respectively, to 71.1 (26.9) and 78.1 (23.1) at week 48 and to 77.0 (25.4) and 81.8 (20.8) at week 108 (all *P* < 0.001 for within-group comparison with baseline). The mean (SD) change from baseline for patients with and without daily headache was increased slightly from week 48 (22.7 [27.0] and 25.7 [25.6], respectively) to week 108 (27.6 [29.7] and 27.0 [26.1]; Fig. 3a and b). There was no significant between-group difference in change from baseline in any mean (SD) MSQ domain at any time point (Fig. 3).

### Safety and tolerability

OnabotulinumtoxinA was well tolerated in patients with and without daily headache (Table 2). AEs occurred in 99 patients (71.7%) with daily headache and 297 (58.9%) without daily headache. A total of 27 patients (4.2%) experienced AEs that led to study discontinuation: 10 (7.2%) of those with daily headache and 17 (3.4%) of those without daily headache at baseline. Serious AEs occurred in 20 patients (14.5%) with daily headache and 44 (8.7%) without daily headache. Serious AEs included migraine (*n* = 5 [0.8%]), suicidal ideation (*n* = 4 [0.6%]), and noncardiac chest pain, malignant melanoma, and headache (all *n* = 3 [0.5%]); we did not observe any clear differences between those with and without daily headache. Only 1 serious AE was considered to be treatment-related (generalized rash), which occurred in the daily headache subgroup.

**Table 2:** Summary of Adverse Events in Patients With and Without Daily Headache

AE, n (%)	With Daily Headache (*n* = 138)	Without Daily Headache (*n* = 504)
≥1 AE	99 (71.7)	297 (58.9)
Serious AE	20 (14.5)	44 (8.7)
Study discontinuation due to AE	10 (7.2)	17 (3.4)
≥1 TRAE	24 (17.4)	97 (19.2)
Individual TRAEs occurring in ≥1% of any subgroup		
Neck pain	5 (3.6)	23 (4.6)
Eyelid ptosis	3 (2.2)	14 (2.8)
Musculoskeletal stiffness	4 (2.9)	13 (2.6)
Injection site pain	3 (2.2)	11 (2.2)
Headache	2 (1.4)	8 (1.6)
Muscular weakness	1 (0.7)	9 (1.8)
Facial paresis	1 (0.7)	6 (1.2)
Migraine	0 (0.0)	6 (1.2)
Skin tightness	1 (0.7)	5 (1.0)
Influenza-like illness	2 (1.4)	1 (0.2)
Balance disorder	2 (1.4)	0 (0.0)
Dizziness	3 (2.2)	0 (0.0)

*AE* adverse event, *TRAE* treatment-related adverse event

Treatment-related AEs occurred in 24 patients (17.4%) with daily headache and 97 (19.2%) without daily headache. Treatment-related AEs occurring in ≥2% of either subgroup included neck pain, eyelid ptosis, musculoskeletal stiffness, and injection site pain (Table 2).

## Discussion

The primary analysis of the COMPEL Study data showed that onabotulinumtoxinA 155 U, when administered for preventive treatment of CM according to the fixed-dose, fixed-site injection paradigm over 9 treatment cycles (108 weeks), was associated with reductions in headache-day frequency and improvement in a range of other efficacy measures and had a favorable tolerability profile. These findings replicated and extended the findings of the earlier PREEMPT studies [7, 9, 10]. Our results support the usefulness of onabotulinumtoxinA for reducing headache days and disability and improving quality of life for up to 108 weeks (9 treatment cycles) in people with CM with daily headache; however, a longer duration of treatment was required to fully realize the treatment effect on headache in patients with daily headache versus those without daily headache. No new safety concerns were identified.

In the early stages of treatment, onabotulinumtoxinA was associated with a significantly smaller reduction in headache days and moderate to severe headache days in patients with daily headache than in those without daily headache. However, by week 108 (after 9 treatment cycles), onabotulinumtoxinA improved headache day and moderate to severe headache-day frequency to a similar degree in patients with daily headache and those with no daily headache, providing clinically useful information to support the management of CM in this challenging subgroup of CM. In our study, people with daily headache were less likely to complete the study than people without daily headache. This could be in part because people with CM and with a high frequency of headaches are reported to distrust treatment, partially because of the inability of treatment to prevent further migraine attacks [16]. Thus, our finding that after 9 treatment cycles, onabotulinumtoxinA reduced headache-day frequency and moderate to severe headache-day frequency to a similar level in patients with CM and daily headache as in those without daily headache is important. People with migraine do not expect that their pain can be completely controlled but do expect their healthcare professionals to provide realistic information about the prospects of treatment [16]. Our results enable healthcare professionals to provide people with CM with daily headaches evidence-based advice about the time taken to achieve optimal therapeutic benefit and to encourage persistence with onabotulinumtoxinA treatment.

The effect of preventive treatment on quality of life and migraine-related disability is also important from a patient’s perspective [17]. In addition to efficacy measures focused on headache frequency, it is recommended that the effect of preventive treatment on disease-related disability and health-related quality of life be assessed using validated tools [18]. It is suggested that a > 5-point change in HIT-6 scores represents a clinically meaningful change [19]. In patients with or without daily headache, onabotulinumtoxinA treatment was associated with a ≥ 5-point change from baseline in HIT-6 total scores from week 24 through week 108. Similarly, in patients with or without daily headache at baseline, MIDAS scores were reduced by approximately 40 points by week 60, and these reductions were maintained throughout the study. When considered with the results from the other measures of migraine-related disability and quality of life we assessed, these results suggest that the reduction in headache frequency observed in the COMPEL Study would be clinically meaningful to patients with CM, including those with daily headache at baseline.

For the MSQ Role Preventive domain, a 5- to 8-point improvement in scores indicates a clinically meaningful response, whereas for the MSQ Role Restrictive and MSQ Emotional Function domains, 5-point and 8- to 10-point improvements, respectively, are considered clinically meaningful [20]. In our study, regardless of daily headache status at baseline, onabotulinumtoxinA treatment was associated with a clinically meaningful increase in all MSQ domain scores (Role Preventive increased by approximately 15–20 points; Role Restrictive and Emotional Function both increased by approximately 25 points).

Physicians and patients alike seek preventive treatments that not only reduce headache-day frequency but also lessen overall migraine-related disability [17]. In patients with CM and daily headache, a subgroup that has not typically been evaluated in clinical trials [5, 6, 21, 22], we found that onabotulinumtoxinA not only reduced headache-day frequency after 9 treatment cycles but also reduced migraine-related disability and improved health-related quality of life. Our results suggest that a longer period of treatment with onabotulinumtoxinA may be required to produce its maximal effect on headache-day reduction in patients with daily headache than in those without daily headache. For patients with daily headache, multiple treatment cycles for ≥108 weeks may be required to ensure these patients fully realize the benefits of onabotulinumtoxinA treatment. However, as early as the second treatment cycle, these patients experienced a reduction in the frequency of headache days and moderate to severe headache days.

### Study limitations and strengths

As an open-label study, the COMPEL Study is useful for gaining additional information about the long-term use of onabotulinumtoxinA now that safety and efficacy have been established in the PREEMPT studies [10]. Nonetheless, there are inherent limitations associated with open-label studies. One such limitation is the absence of a placebo control group to enable treatment comparisons. In addition, in studies with long-term follow-up, such as the COMPEL Study, loss to follow-up is inevitable and may have an effect on treatment outcomes. Furthermore, concomitant medication use may change over the duration of the study [11]. These limitations have been discussed in more detail previously [12]. A completer analysis demonstrated only slightly better results (a greater reduction in headache days from baseline than the primary analysis using mLOCF: − 11.8 vs − 10.7 days) despite low persistency rates, further supporting the validity of the COMPEL Study.

In addition, the fluctuations in headache-day frequency over time that occur in people with CM can make it difficult to interpret study results [23]. It is recommended that primary endpoints for headache day outcomes be based on prospective diary data [18], as in the COMPEL Study. Furthermore, it is recommended that health-related quality of life and disability be assessed using validated disease-specific tools [18]. HIT-6 and MSQ have been validated for use in CM [24, 25]. Although the MIDAS questionnaire has been validated for use only in migraine [14], it is likely to also be valid for CM [18]. Nonetheless, given the open-label nature of the study and subjective reporting by enrolled patients [18], the results of this subanalysis should be interpreted cautiously.

Despite the potential limitations discussed above, the reduction in headache frequency from baseline in the analysis population from the COMPEL Study at week 24 parallels that from the double-blind, placebo-controlled phase of the PREEMPT studies (− 7.4 vs − 8.4 days) [9, 12]. Similarly, results at week 24 for HIT-6 and week 48 for MSQ scores are comparable with those reported at week 24 in the PREEMPT studies (HIT-6, − 4.8; MSQ Role Preventive, + 13.1; MSQ Role Restrictive, + 17.0; MSQ Emotional Function, + 17.9) [9], supporting the relevance of the COMPEL Study results and, by extension, this subanalysis of COMPEL data.

## Conclusions

Data from the COMPEL Study support the sustained benefit and tolerability of onabotulinumtoxinA for up to 108 weeks (9 treatment cycles) in patients with CM with daily headache at baseline. OnabotulinumtoxinA treatment was associated with a reduction in headache-day frequency and improvement in disability and quality of life in patients with and without daily headache at baseline. Although patients with daily headache may need to continue treatment with onabotulinumtoxinA for up to 9 cycles to experience maximal benefit in headache-day reduction, clinically meaningful reductions in migraine-related disability and quality of life occur earlier. No new safety concerns were identified, and onabotulinumtoxinA appeared to be well tolerated in patients with daily headache at baseline.

## Additional file

Additional file 1: **Figure S1.** Effect of onabotulinumtoxinA on (A) headache day frequency and (B) moderate/severe headache day frequency in patients with and without daily headache at baseline. (PDF 32 kb)

## Abbreviations

AE: Adverse event
CM: Chronic migraine
COMPEL: Chronic migraine OnabotulinuMtoxinA Prolonged Efficacy open-Label
HIT-6: 6-item Headache Impact Test
MIDAS: Migraine Disability Assessment
mLOCF: Modified last observation carried forward
MSQ: Migraine-Specific Quality-of-Life Questionnaire
PREEMPT: Phase III REsearch Evaluating Migraine Prophylaxis Therapy
