# Supplementary material for: Effects of onabotulinumtoxinA treatment in chronic migraine patients with and without daily headache at baseline: results from the COMPEL Study
Source: J Headache Pain. 2019 Feb 1;20(1):12. doi: 10.1186/s10194-018-0953-0 (PMC6734507; doi:10.1186/s10194-018-0953-0)
Supplement: Supplementary file 1 — Figure S1. Effect of onabotulinumtoxinA on (A) headache day frequency and (B) moderate/severe headache day frequency in patients with and without daily headache at baseline. (PDF 32 kb) [file 10194_2018_953_MOESM1_ESM.pdf]

Supplementary Figure 1: Effect of onabotulinumtoxinA on (A) headache day frequency and (B) moderate/severe headache day frequency in patients with and without daily headache at baseline.

A

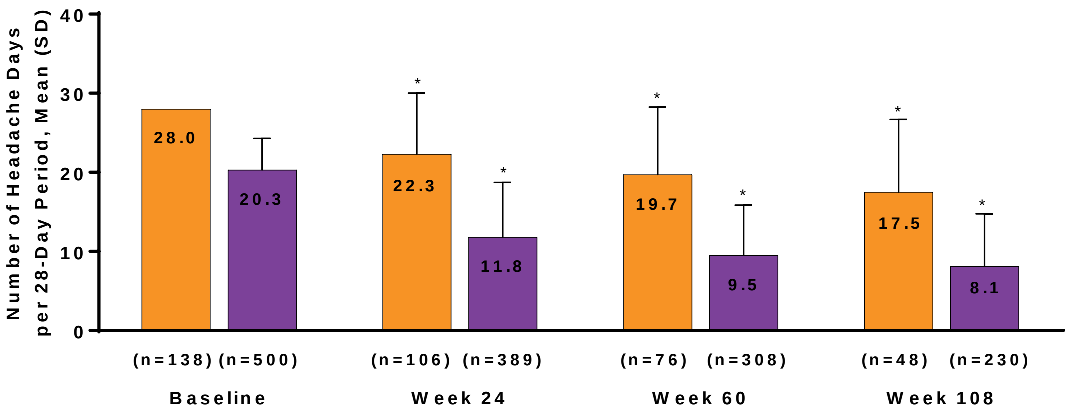

B

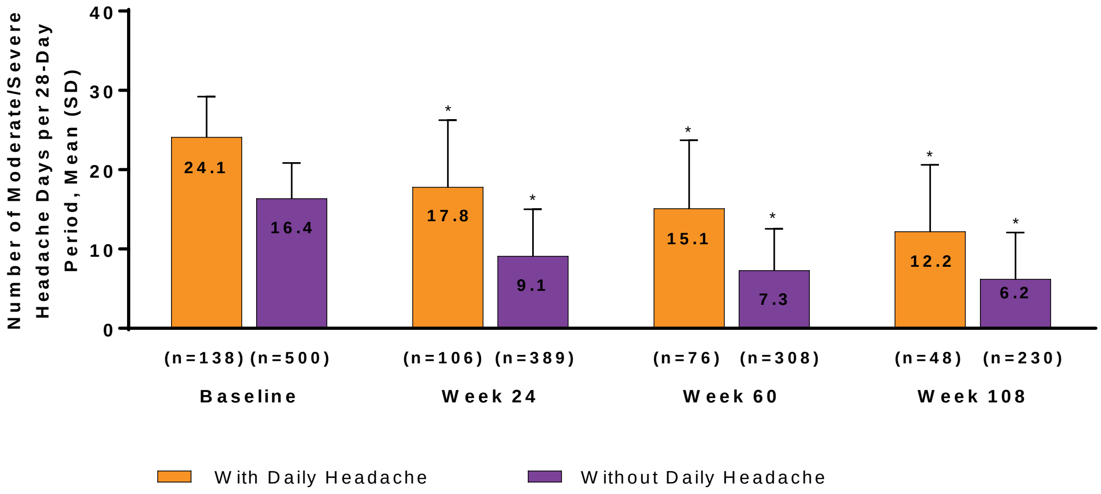

*P<0.001 for within group comparison with baseline.
